# Anaesthetic Management of a Labrador Retriever Undergoing Adrenalectomy for Phaeochromocytoma Excision, a Case Report

**DOI:** 10.3389/fvets.2022.789101

**Published:** 2022-03-16

**Authors:** Ekaterina Gámez Maidanskaia, Claudia Spadavecchia, Simona Vincenti, Alessandro Mirra

**Affiliations:** ^1^Anaesthesiology and Pain Therapy Division, Department of Clinical Veterinary Medicine, Vetsuisse Faculty, University of Bern, Bern, Switzerland; ^2^Surgery Division, Department of Clinical Veterinary Science, Vetsuisse Faculty, University of Bern, Bern, Switzerland

**Keywords:** case report, phaeochromocytoma, urapidil, magnesium sulphate (MgSO_4_), hypertension, hypotension

## Abstract

Perioperative management of cases undergoing phaeochromocytoma removal should aim at normalising blood pressure and heart rate, restoring volume depletion, and preventing catecholamine release induced by surgical manipulation. In this case report, a novel pharmacological approach in a dog undergoing surgical tumour excision is described. A 7-year-old 25-kg spayed female Labrador Retriever presented for repeated episodes of generalised weakness, pale mucous membranes, tachycardia, tremor, panting, vomiting, and hypertension over the last month was referred for surgical treatment of a left-sided adrenal tumour with invasion of the caudal vena cava. Severe hypertensive episodes occurred repeatedly, starting early during the anaesthetic period, while clipping and cleaning the abdominal area, and continued intraoperatively when the tumour was handled. Moderate hypotension occurred once the tumour was isolated and worsened during temporary caudal vena cava flow interruption and cavotomy. The patient was treated preoperatively with phenoxybenzamine to prevent hypertensive crises. Intraoperatively, magnesium sulphate and urapidil were used to control blood pressure. This treatment was effective in reducing the magnitude of blood pressure spikes but not sufficient to prevent hypertensive peaks, especially during tumour manipulation. Hypotension was treated with synthetic colloid and crystalloid boli, and noradrenaline continuous infusion. Blood transfusion was performed in response to acute bleeding during cavotomy. The dog recovered successfully from anaesthesia and its quality of life was deemed excellent by the owner at the last follow up, 22 months after surgery. The histopathology confirmed the diagnosis of phaeochromocytoma with an invasion of the phrenicoabdominal vein. In the present case, we obtained a successful outcome but failed to provide haemodynamic stability throughout the procedure.

## Introduction

Phaeochromocytomas are catecholamine-producing neoplasia arising from the chromaffin cells of the adrenal medulla (1–3). In dogs, although uncommon (0.1% of all tumours) (4), it is the second most diagnosed adrenal tumour after adenocarcinoma (5). Clinical signs are non-specific and result from the excessive production of catecholamines (4, 6). Adrenalectomy is usually the treatment of choice.

According to Connor et al. (1) and Lenders et al. (7), normalisation of blood pressure and heart rate, restoration of depleted volume, and gentle manipulation of the tumour to prevent catecholamine release are the cornerstones of PCC perioperative management (1, 7). Uncontrolled catecholamine release can lead to further complications as cardiac arrhythmias or pulmonary oedema, among others (1, 8); thus, it must be prevented or treated to improve the survival rate (3). Hypotension and haemorrhage are also potential complications to be addressed during anaesthesia in dogs (1, 4, 5, 8). Pulmonary embolism should be considered as a possible perioperative complication (5).

## Case Presentation

A 7-years-old, spayed female Labrador Retriever weighing 25-kg was referred to the veterinary teaching hospital of the University of Bern, for a second opinion concerning a left-sided adrenal mass.

The referring veterinarian described repeated episodes of generalised weakness, pale mucous membranes, tachycardia, tremor, panting, and vomiting during the previous month. Computerised tomography images revealed a left-sided adrenal gland tumour, invading the caudal vena cava (CdVC). According to the results of the endocrinological tests (negative urinary metanephrines and ACTH stimulation) and a lack of recorded hypertensive episodes, an adrenal adenocarcinoma was suspected. However, due to the invasion of the CdVC and the clinical signs, phaeochromocytoma (PCC) could not be ruled out. Surgical removal of the mass was advised. Phenoxybenzamine (PBZ) was started by the referral veterinarian at 0.6 mg/kg twice per day and was maintained at this dose until ~36 h before surgery (14 days). The dose was not titrated based on blood pressure measurement.

On the day of surgery, the dog presented a heart rate (HR) of 88 beats per minute (BPM), a respiratory rate of 26 breaths per minute, a rectal temperature of 38.2°C, pink and moist mucous membranes and a capillary refill time of 1.5 s. Oscillometric blood pressure was 124/86 (99) [SAP/DAP(MAP)] mmHg. Lung and heart auscultation were unremarkable. Dog erythrocyte antigen was 1.1+, and blood cross-matching was performed before anaesthesia. Complete blood count, biochemistry, prothrombin, and partial thromboplastin times were within normal values. Baseline magnesium was 0.7 mmol/L. The patient was not treated preoperatively with heparin due to the risk of bleeding.

### Perioperative Anaesthetic and Analgesic Management

Methadone (0.2 mg/kg IV) was administered in premedication, inducing slight sedation. Preoxygenation was performed using 100% “flow by” oxygen, at 4 L/min, over 5 min. Lidocaine (2 mg/kg IV), fentanyl (0.005 mg/kg IV), and propofol (total of 8 mg/kg IV) were used to induce general anaesthesia. The trachea was intubated with an 11-mm internal diameter, low volume, high pressure, silicone endotracheal tube, then connected to an adult anaesthetic circle system. The patient was allowed to breathe spontaneously and a stable depth of anaesthesia (absence of palpebral reflex, ventromedial eye rotation, and absence of jaw tone) was targeted using isoflurane (Fe 1–1.3%) in oxygen combined with IV lidocaine and fentanyl continuous rate infusions (CRI) (starting rates 1.8 and 0.007 mg/kg/h, respectively). The left cephalic vein was catheterized using an 18-G over the needle catheter to administer emergency drugs and transfusion if needed.

The lumbosacral area was clipped and aseptically prepared. Preservative-free morphine (0.1 mg/kg) and methadone (0.1 mg/kg) mixed with 0.2 mL/kg sterile saline solution were administered epidurally in the lumbosacral space, using a spinal needle (no Tuohy needle was available). Correct needle placement was confirmed by a positive hanging drop test and lack of resistance to injection.

Overall procedure monitoring consisted of a three-lead ECG, transmittance pulse oximetry, side-stream capnography with agent analyzer and oesophageal temperature, non-invasive oscillometric arterial blood pressure, and invasive arterial blood pressure (IABP). The patient was positioned in dorsal recumbency, and the right dorsal pedal artery was catheterized using a 22-G over-the-needle catheter but not connected to the transducer because during abdominal scrubbing the heart rate dropped from 56 to 34 BPM. Some second-degree atrioventricular blocks were observed; no non-invasive arterial blood pressure value was displayed at that moment. The recumbency was changed to a right lateral and a bolus of glycopyrrolate (0.01 mg/kg) was administered IM. The IABP transducer was placed at the level of the patient's heart and zeroed at atmospheric pressure. The IABP line was connected to the arterial catheter and the fast-flush test showed no under or overdamping. The monitor displayed a systolic/diastolic (mean) arterial pressure [SAP/DAP(MAP)] of 178/77(105) mmHg, respectively. The heart rate increased to 62 BPM and AV blocks disappeared within 10 min from glycopyrrolate administration; at this point, the patient was repositioned in dorsal recumbency. Five minutes later, an ultrasound-guided bilateral subcostal and lateral transversus abdominis plane block was performed, administering 0.1 mL/kg of sterile ropivacaine (0.5%) diluted 1:1 with sterile sodium chloride per each of the 4 injection points (9).

In the surgery room, the dog was placed in dorsal recumbency. Blood pressure (BP) was 133/68(84) mmHg and HR 85 BPM; both remained stable (SAP 131 ± 1, DAP 67 ± 1, MAP 82 ± 1, and HR 85 ± 4 BPM; mean ± SD) until the mass was manipulated. Active warming was provided using an electronic warm water pad set to 38°C and warm air set at 40°C, targeting an oesophageal temperature of 37.5–38.5°C. Approximately 40 min before the occlusion of the CdVC, active cooling was started by switching off warming devices and surrounding the patient with ice blocks packed in paper towels. A temperature reduction of around 2 degrees (from 37 to 35.1) was achieved by the end of the occlusion period.

The patient was allowed to breathe spontaneously for the first 115 min; thereafter pressure-controlled ventilation was started due to the increasing end-tidal CO_2_ (EtCO_2_). Peak inspiratory pressure was maintained between 9 and 13 cmH_2_O, and no positive end-expiratory pressure was set up. The respiratory rate varied between 18 and 40 breaths per minute and tidal volume between 7 and 13 mL/kg targeting an EtCO_2_ of 35–45 mmHg.

After median laparotomy associated with left and right paracostal approaches, a 5 cm large left adrenal mass was located, strongly attached to the surrounding structures. Moreover, a 10 cm long and 4 cm large invasion of the CdVC through the left phrenicoabdominal vein was found. Three Rumel tourniquets were placed (cranial to the tumour thrombus on the pre-hepatic CdVC, caudal to the left phrenicoabdominal vein on the CdVC and on the left renal vein). Despite careful adrenal mass manipulation, several hypertensive crises occurred. A total of 7 SAP spikes between 150 and 200 mmHg and 14 SAP spikes ≥200 mmHg were recorded, with the highest value being 394 mmHg.

**Figure 1:**
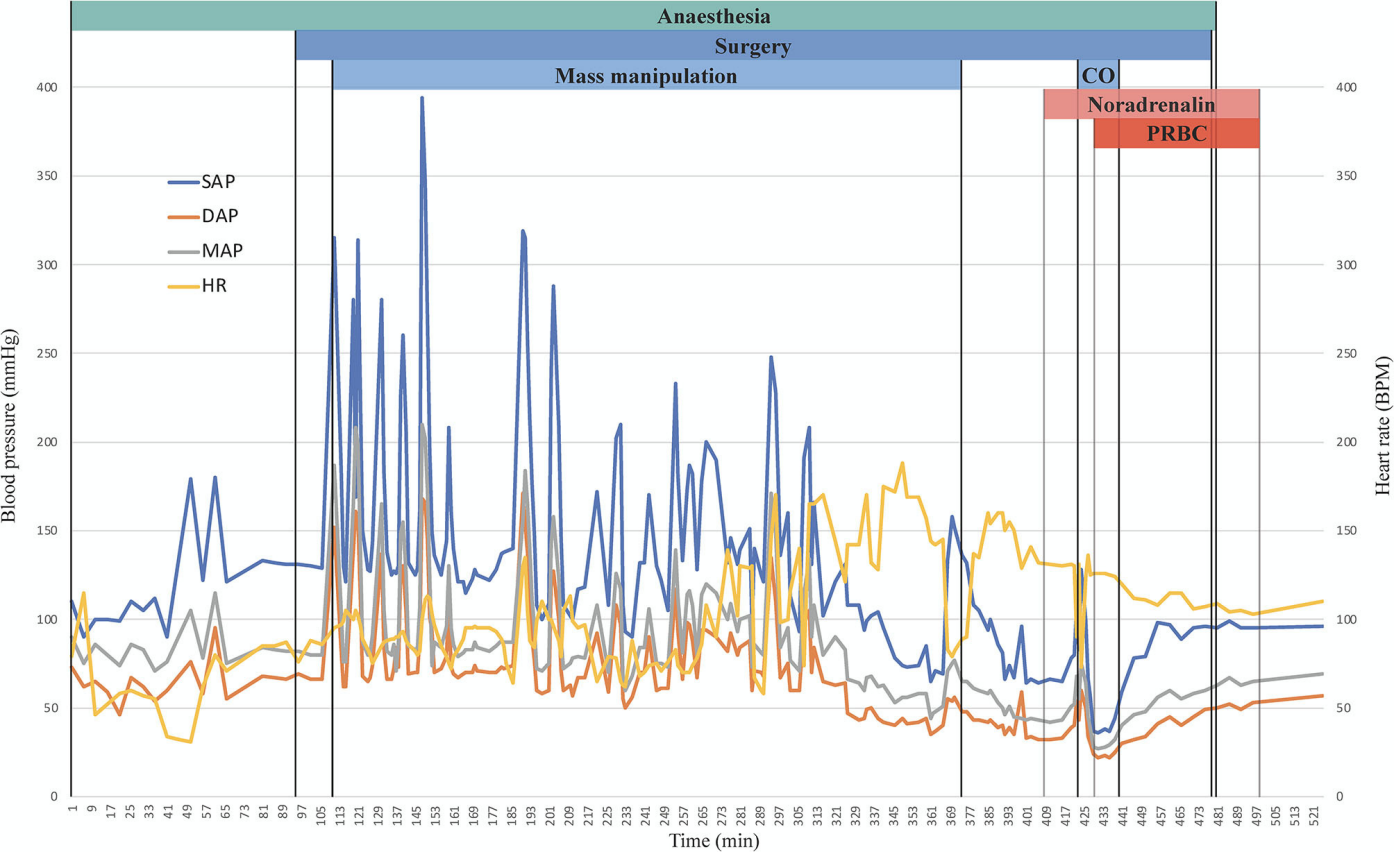
Graphical representation of systolic (blue), diastolic (orange), and mean (grey) arterial pressure and heart rate (yellow) over time expressed in minutes after induction. On the top of the graph, a simplified timeline of the procedure is depicted, the end of the graph corresponds to the extubation. SAP, systolic arterial pressure; DAP, diastolic arterial pressure; MAP, mean arterial pressure; BPM, beats per minute; CO, Caval occlusion; PRBC, packed red blood cells.

**Figure 2:**
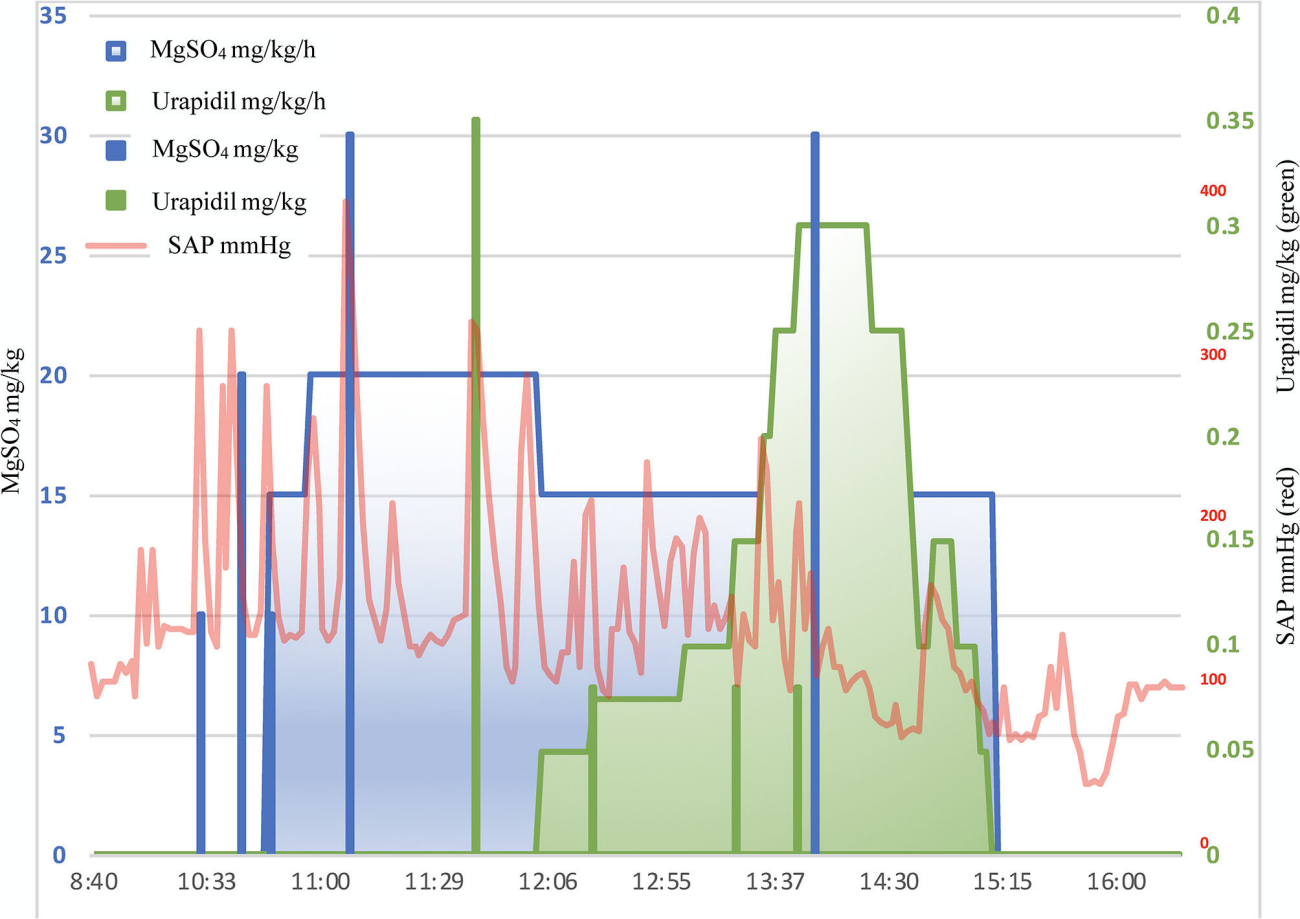
Graphical representation of MgSO_4_ (blue) and urapidil (green) administration plotted against SAP (red) over time.

The hypertensive crises were treated first with magnesium sulphate (MgSO_4_) alone and afterwards combined with urapidil. The haemodynamic evolution thorough the procedure and the pharmacological management of the hypertensive crisis are depicted in more detail in Figures 1, 2, respectively. A total of 3 boli (10, 20, and 10 mg/kg IV) of MgSO_4_ were administered before CRI was started at 15 mg/kg/h. Approximately 1 h later, venous blood was sampled for blood gas analysis and magnesium quantification. The results showed the presence of respiratory acidosis, with a pH of 7.149 and an arterial pressure of CO_2_ (P_a_CO_2_) of 68.5 mmHg. The gap between P_a_CO_2_ and EtCO_2_ was 18.5 mmHg. Magnesium blood concentration was 1.66 mmol/L (physiological range 0.63–0.96 mmol/L). Thus, the rate of MgSO_4_ was reduced and the first bolus of urapidil was administered at 0.35 mg/kg IV, followed by a CRI at 0.05 mg/kg/h, further increased during the procedure up to 0.3 mg/kg/h.

Boli of magnesium sulphate (total of 4) allowed to achieve an average reduction in SAP of 61 ± 5% (from 325.75 ± 48.3 to 124.75 ± 2.6 mm Hg). Similarly, after urapidil boli (total of 4), the average reduction was 59 ± 6% (from 222 ± 70.2 to 94 ± 4.2 mm Hg). Both MgSO_4_ and urapidil infusions maintained a SAP of 129 ± 21 mmHg but did not prevent BP spikes, and boli were needed to punctually treat them.

Two ventricular tachycardia episodes occurred during mass manipulation. Both had a good response to 2 mg/kg lidocaine bolus IV. After the first episode, the lidocaine CRI was increased to 3 mg/kg/h. No further arrhythmias were seen during the whole procedure.

A steady decrease in BP occurred as the tumour was getting surgically isolated. Thus, urapidil infusion was decreased stepwise and finally interrupted, alongside with MgSO_4_. As the MAP reached values below 60 mmHg, three boli of crystalloids (10 mL/kg each one along 10 min) and two boli of synthetic colloid (5 mL/kg each one along 10 min) were administered. Twenty minutes after tumour isolation, the MAP decreased to <50 mmHg. At this point, lidocaine CRI was reduced to 1.8 mg/kg/h and noradrenaline (NA) started at 0.0005 mg/kg/min.

Then, Rumel tourniquets were tightened to temporarily interrupt the caval flow. During the tumour removal, the cranial tourniquet broke causing rapid haemorrhage, which was promptly stopped by placing large Satinsky clamps on the venotomy site. One unit of packed red blood cells was administered (no quantification of blood loss was performed), while isoflurane, fentanyl and lidocaine infusions were discontinued due to a sudden marked decrease in blood pressure to 36/22 (27) mmHg. Crystalloids were given at 1,200 mL/h for 20 min while NA CRI was gradually increased reaching 0.005 mg/kg/min, then maintained until the end of the procedure. Total (Rumel tourniquets) and partial (Satinsky clamps) caval flow occlusion lasted 5 and 10 min, respectively; BP values registered during this period were SAP 54 ± 21, DAP 29 ± 9, and MAP 37 ± 12 mm Hg, and HR value was 125 ± 4 BPM (mean and ±SD). Once the caval flow was restored BP was 59/30 (40) mmHg and it continued increasing until it reached 95/45 (58) mmHg; then, isoflurane, fentanyl and lidocaine were restarted.

A total of 6 blood gas analyses (two venous and four arterial) were performed during surgery (Table 1). Severe respiratory acidosis and a large P_a_CO_2_-EtCO_2_ gap (between 12.4 and 25.3 mmHg) persisted. The P_a_O_2_/FiO_2_ ratio was ranging between 250 and 473 mmHg in the arterial samples.

**Table 1:** The table summarises six intraoperative and two postoperative blood gases performed.

**Time point (min)**	**Sample**	**pH**	**P_**a**_CO_**2**_ (mmHg)**	**EtCO_**2**_ (mmHg)**	**PaO_**2**_ (mmHg)**	**HCO_**3**_ (mmol/L)**	**BE (mmol/L)**	**sO_**2**_ (%)**	**Glu (mmol/L)**	**Lac (mmol/L)**	**SpO_**2**_ (%)**	**FiO_**2**_ (%)**	**P_**a**_O_**2**_/FiO_**2**_ ratio**
164	Venous	7.149	68.5	50	54.4	23.3	−6.5	75.8	8.2	0.38	98	46	–
201	Artherial	7.188	59.4	47	326.3	22.1	−6.9	99.7	9.6	0.61	100	93	351
240	Venous	7.187	62.3	37	55.5	23.1	−5.9	80.1	9.7	0.68	98	95	–
325	Artherial	7.094	64.5	45	168.7	19.3	−11.2	99.6	14.7	1.63	97	58	291
398	Artherial	7.151	57.6	36	136.9	19.7	−9.6	98.7	15.1	3.69	98	55	250
487	Artherial	7.132	60.3	42	452.7	19.7	−9.7	99.7	11.2	3.37	100	96	472
1,090	Artherial	7.329	40.6		79.2	20.9	−4.7	95.9	6	1.12		^*^27	293
1,330	Artherial	7.308	35.8		80.3	17.5	−7.9	95.5	4.8	2.53		^*^27	297

* *Inspired fraction of oxygen is estimated based on Jagodich et al. (36), considering 0.1 mL/kg/h flow of 100% O_2_ administered through a unilateral 10 Fr nasal canula. P_a_O_2_/FiO_2_ ratio calculated according to Calabro et al. (37). Time points are expressed in minutes considering induction as time point 0*.

When the surgery was finished, isoflurane was discontinued while lidocaine and fentanyl CRIs were maintained for the postoperative period at 1.8 and 0.005 mg/kg/h, respectively. Noradrenaline CRI was reduced by 0.001 mg/kg/min every 5 min from the end of the surgery, and no drop in blood pressure was observed. When 0.002 mg/kg/min was reached, the NA CRI was stopped, and the pressure monitored invasively for ~30 min, until extubation, when BP values were 96/56 (70). After extubation and transferred to the intensive care unit. One and five hours postoperatively, arterial blood gas values showed a decrease in P_a_CO_2_ from 60 mmHg (last intraoperative measurement) to 40 and 35 mmHg, respectively (Table 1). Duration of surgery and anaesthesia was longer than expected due to several complications (e.g., caval Rumel tourniquet rupture, bilateral paracostal approach), the severity of the tumour invasion in the surrounding tissues (slow and gentle handling) and substantial anaesthetic instability. Anaesthesia and surgery lasted 490 and 325 min, respectively.

### Outcome and Follow-Up

**Figure 3:**
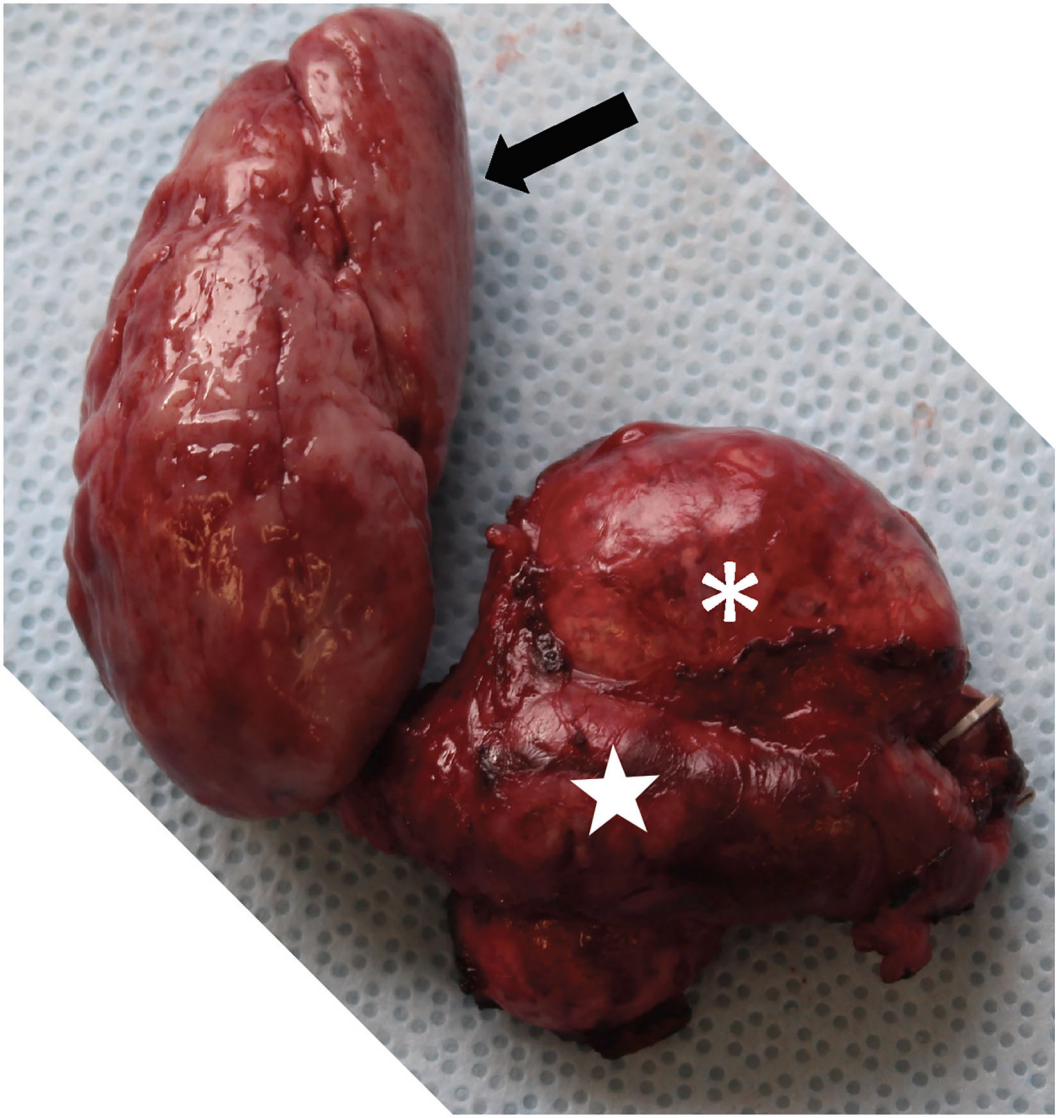
Picture showing the pheochromocytoma after complete removal. Note the left adrenal mass (white asterisk), the severely distended left phrenicoabdominal vein (white star) and the tumour thrombus that extended into the CdVC for 10 cm (black arrow).

No evidence of renal damage was detected on postoperative blood work performed on day 1 and 4 after surgery. The patient did not show any visual impairments during hospitalisation although no direct control of the retina was performed. The dog was discharged in good clinical condition 5 days after surgery. The histopathology of the mass revealed a phaeochromocytoma (Figure 3). On a telephonic follow up 22 months after surgery, the owner reported an excellent quality of life of the dog.

## Discussion

The anaesthetic management of dogs undergoing phaeochromocytoma excision raises a true challenge. The rare clinical presentation and the absence of controlled randomised trials, the unpredictable and life-threatening haemodynamic fluctuations and the need for continuous and thorough monitoring to detect them, makes it particularly difficult to deal with these cases.

In human medicine, the preoperative administration of alpha-receptor blockers in PCC is suggested (low quality of evidence) since 2014, even in normotensive patients, in the attempt to prevent perioperative haemodynamic instabilities (1, 10). In veterinary medicine, preoperative PBZ is recommended for patients affected by PCC (2, 11–15). Described durations of treatment range from 7 to 120 days (3). Phenoxybenzamine is a non-competitive, irreversible, long-acting, non-selective α-blocker (1, 3, 10), suggested to minimise the deleterious effects of excessive catecholamine secretion that might occur during the perioperative period (3). Its administration for 10–14 days has been suggested to help restore the circulating blood volume, depleted due to arteriolar and venous constriction after long exposure to the high concentration of circulating catecholamines (8) and is the only medication in veterinary management of PCC that has been described to reduce perioperative mortality (3). However, administration of PBZ has no influence on the appearance of intraoperative arrhythmias in dogs (3) and can induce reflex tachycardia due to inhibition of presynaptic α-2 blockade, which often requires pre-operative β-blockers in humans (10). A human paediatrics study showed that, despite the use of PBZ for 4 days preoperatively, around 70% of the treated cases presented rises in SAP > 180 mmHg (16). As described in human and veterinary literature (1, 3, 12–15, 17, 18), PBZ was started preoperatively by the referral veterinarian and maintained for 14 days. To avoid intra- and postoperative refractory hypotension, PBZ was withheld for 36 h preoperatively (1). This may have allowed alpha receptors resynthesis and influenced intraoperative haemodynamic instability. Moreover, the lack of preoperative PBZ titration according to serial BP measurements could have led to a suboptimal administration of the drug.

In the veterinary literature, intraoperative hypertension in PCC is treated using drugs such as phentolamine (3, 15) sodium nitroprusside (15, 19, 20), esmolol (14), acepromazine (11, 14), and higher % of inhalants (11, 14), alone or in combination. Tachycardia is generally treated with esmolol (14, 15). In our case, neither phentolamine nor sodium nitroprusside were available; thus, MgSO_4_ and urapidil were used.

The administration of MgSO_4_ to control catecholamine-induced crises has been already described in human medicine two decades ago (21) and it has been used to control hypertension in PCC resection cases (1, 18, 22, 23). In the veterinary literature, only one retrospective study reported the use of MgSO_4_ to control hypertensive crises caused by PCC (3). Magnesium sulphate inhibits the release of catecholamines from the adrenal medulla and reduces the sensitivity of alpha-adrenergic receptors to catecholamines, besides exerting a direct vasodilator effect (1, 21) *via* its endogenous calcium antagonism (1, 23). In the present case, MgSO_4_ boli were effective but short-lasting (7.5 ± 0.5 min from the start of injection until a new pressure rise). The use of a continuous infusion did not prevent hypertensive peaks even in combination with urapidil. Magnesium should be used with caution, monitoring its plasmatic concentration, ECG activity and BP (24). Some of the hypotensive episodes observed may have been also exacerbated by MgSO_4_ administration.

In humans, urapidil showed effective prevention of intraoperative hypertensive spikes and postresection/postoperative hypotension when administered as an infusion during the three preoperative days and continued during surgery until surgical clamp of the adrenal vein (10). To the authors' knowledge, the clinical use of this drug has not been reported in veterinary medicine before. Urapidil is a short-acting (half-life 2.7 h) peripheral and central α-1 adrenoreceptor antagonist, therefore causing vasodilation (10, 25–27). It is also an antagonist of the central serotonin receptors inducing an additional reduction in adrenergic activity (26). In contrast to PBZ, its specific α-1 inhibition and serotonin receptors antagonism, weakly blocking beta receptors, do not induce tachycardia, which could prevent the use of beta-blockers (10). As mentioned before, urapidil in combination with MgSO_4_ helped in reducing the magnitude of BP spikes but did not prevent further hypertensive peaks. Moreover, it could have also contributed to the postresection hypotension. The perioperative infusion of urapidil suggested by Tauzin-Fin et al. (10) could be considered as a possible alternative option to PBZ in veterinary medicine.

Dexmedetomidine, an α-2 receptor agonist, has been described in human medicine in combination with other drugs (e.g., MgSO_4_) to achieve haemodynamic stability during phaeochromocytoma resection (1, 28–30). The activation of α-2 receptors leads to a dose-dependent reduction in plasma catecholamines since they take part in the regulation of noradrenaline release through a negative feedback mechanism (31). However, bradyarrhythmias have been described during dexmedetomidine use (32, 33). Perioperative dexmedetomidine CRI was originally foreseen but due to the drop in HR (most probably baroreceptor mediated) and presence of second-degree atrioventricular blocks, its administration was finally avoided, even though its administration could have helped in treating the subjacent cause.

As catecholamine release can be exacerbated by sympathetic stimulation due to pain, a multimodal analgesic approach was used. Fentanyl and lidocaine infusions, an epidural administration of opioids, and a transversus abdominis plane block with ropivacaine were performed. These analgesic strategies alone or combined have been previously described in the veterinary literature for adrenalectomies (5). Even if the co-administration of methadone and morphine is not based on scientific evidence, methadone was used expecting a faster onset due to its higher lipophilicity, thus ensuring intraoperative analgesia, while morphine was used to cover postoperative pain.

Hypotension is also listed as a common complication in PCC excision (3). Once the adrenal gland is removed, severe hypotension may happen due to residual action of vasodilators and catecholamine withdrawal (1, 18). In our case, pharmacological support was eventually needed to elevate blood pressure (34). The use of crystalloids, colloids and vasopressors (phenylephrine, ephedrine, dopamine, or dobutamine) has been reported in dogs with PCC (13, 15). To face hypotension, we administered boli of crystalloids and colloids (natural and synthetic), reduced or stopped drug infusions and inhalant administration, and initiated NA. Despite this, the patient remained moderately hypotensive once the tumour was isolated, and severely hypotensive during caval occlusion. The cause of hypotension is most likely multifactorial including haemorrhage, acidosis, mild to moderate hypothermia, and residual effect of vasodilators. The patient returned normotensive immediately after recovery.

Active cooling was performed in an attempt of protecting vital organs from reduced oxygen delivery during temporary occlusion of the CdVC (3). However, hypothermia might promote hypocoagulation and sequestration of platelets in the spleen, facilitating blood losses, and its diuretic effects might contribute to hypotension (35). Nevertheless, since the hypothermia level reached in the presented case was mild, it is difficult to estimate how it might have affected both organ protection and side effects development. The active cooling could have been started earlier in the timeline to achieve lower temperature at the time of caval occlusion.

Due to the high P_a_CO_2_-EtCO_2_ difference, pulmonary thromboembolism was initially suspected. Pulmonary thromboembolism is one of the most relevant postoperative complications reported in dogs affected by adrenocortical tumours (15), its incidence being particularly high in dogs with neoplastic invasion of the vena cava and PCC (5). During the whole procedure, the P_a_CO_2_-EtCO_2_ gap ranged between 12.4 and 25.3 mmHg. However, after recovery, P_a_CO_2_ decreased substantially while the animal had a physiological breathing rate and pattern, suggesting a gap reduction. Thus, even if the presence of transitory thrombi cannot be ruled out, it seems unlikely. Another possible explanation for this event is a strong catecholamine mediated vasoconstriction or/and atelectasia. Neither recruitment manoeuvres were performed, nor PEEP applied to avoid further cardiovascular impairment.

The described use of glycopyrrolate could be debatable, as the bradycardia with second degree AV blocks may have been a result of hypertension due to a catecholamine burst. Given the sudden and fast decrease in heart rate of the patient, a not confirmed diagnosis of phaeochromocytoma (negative urine metanephrines) and the fact that the most described arrhythmias due to catecholamine release are tachyarrhythmias (1, 3) the authors decided to administer it. To avoid an acute increase in blood pressure it was administered IM.

In the present case, we obtained a successful outcome but failed to provide haemodynamic stability throughout the procedure. Further investigations are warranted to find a multimodal approach to effectively avoid blood pressure increase in response to catecholamine release. The combination of magnesium sulphate with urapidil did not provide continuous haemodynamic stability but was effective in treating blood pressure spikes.

### Take Home Messages

The anaesthetic approach to phaeochromocytoma excision is based on careful perioperative management. Patients should be handled gently to minimise perioperative catecholamine release. Blood typing must be performed to promptly replace lost volume in case of haemorrhage. A combination of vasoactive drugs pre- and intraoperatively could be helpful in providing haemodynamic stability. Postoperatively, close control of cardiovascular status and respiratory parameters is advocated, especially if V/Q mismatch have been noticed during surgery.

In the present case report, the combination of magnesium sulphate with urapidil to treat severe hypertension did not provide continuous haemodynamic stability but was effective in treating blood pressure spikes and finally led to a successful outcome.

## Data Availability Statement

The raw data supporting the conclusions of this article will be made available by the authors, without undue reservation.

## Ethics Statement

Ethical review and approval was not required for the animal study because it is a case report. Written informed consent was obtained from the owners for the participation of their animals in this study.

## Author Contributions

EM was involved in anaesthetic management, data collection, and preparation of the first manuscript. CS was involved in editing and reviewing the draft. SV performed the surgery and was involved in draft reviewing. AM was involved in anaesthetic management, data collection, editing, and reviewing the draft. All authors contributed to the article and approved the submitted version.

## Conflict of Interest

The authors declare that the research was conducted in the absence of any commercial or financial relationships that could be construed as a potential conflict of interest.

## Publisher's Note

All claims expressed in this article are solely those of the authors and do not necessarily represent those of their affiliated organizations, or those of the publisher, the editors and the reviewers. Any product that may be evaluated in this article, or claim that may be made by its manufacturer, is not guaranteed or endorsed by the publisher.

## Abbreviations

BP: Blood pressure
CdVC: Caudal vena cava
CRI: Continuous rate infusion
DAP: Diastolic arterial pressure
EtCO_2_: End-tidal carbon dioxide
HR: Heart rate
IABP: Invasive arterial blood pressure
MAP: Mean arterial pressure
MgSO_4_: Magnesium sulphate
NA: Noradrenaline
°C: degrees Celsius
P_a_CO_2_: Arterial pressure of carbon dioxide
PBZ: Phenoxybenzamine
PCC: Phaeochromocytoma
SAP: Systolic arterial pressure
Fe: Expired fraction.
